# SERS-Based Universal AST: Rapid Treatment Guidance for Blood-Culture Bacteria before Species Identification

**DOI:** 10.1021/acs.analchem.5c04833

**Published:** 2026-02-12

**Authors:** Yin-Yi Han, Yu-Tsung Huang, Dai-Feng Li, Ko-Lun Chen, Yi Chi, Hsin-Mei Tsai, Ho-Wen Cheng, Juen-Kai Wang, Yuh-Lin Wang

**Affiliations:** † Department of Anesthesiology, National Taiwan University College of Medicine, Taipei 100225, Taiwan; ‡ Department of Traumatology, 38006 National Taiwan University Hospital, Taipei 100225, Taiwan; § Division of Laboratory Medicine, Department of Internal Medicine, National Taiwan University Hospital, Taipei 100225, Taiwan; ∥ Institute of Atomic and Molecular Sciences, 71556 Academia Sinica, Taipei 106319, Taiwan; ⊥ Center for Condensed Matter Sciences, National Taiwan University, Taipei 106319, Taiwan

## Abstract

Rapid antimicrobial susceptibility testing (AST) is essential for managing bloodstream infections with high mortality, yet most current methods require prior identification of bacterial species, limiting their speed and applicability. To address these limitations, we developed SERS-Uni-AST, a species-independent platform based on surface-enhanced Raman scattering (SERS) that monitors the metabolic responses of bacteria to antibiotics through label-free detection. This approach eliminates the need for species identification, simplifying the testing workflow, and expands applicability to rare or previously unknown bacterial species while significantly reducing turnaround time. Validated on 191 clinical blood-culture isolates encompassing 43 bacterial species and 7 clinically relevant antibiotics, SERS-Uni-AST achieved 92% categorical agreement with standard testing within 5 h. The method demonstrated robust performance across Gram-positive and Gram-negative bacteria, including ESKAPE pathogens, using a predefined, species-independent decision threshold. By enabling timely, susceptibility-guided antimicrobial therapy, SERS-Uni-AST supports early treatment optimization and aligns with the goals of the Surviving Sepsis Campaign and WHO antimicrobial stewardship initiatives.

## Graphical abstract

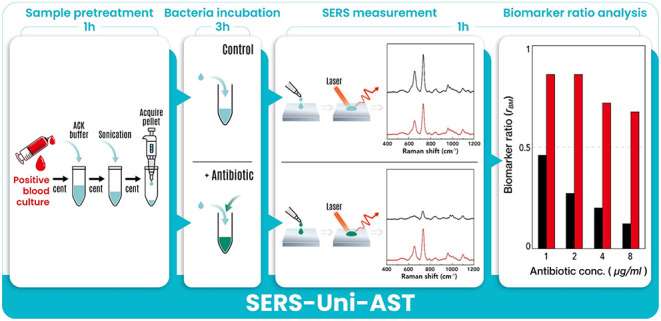

## Introduction

Bacterial bloodstream infection leading to sepsis remains a significant clinical challenge,^1^ with mortality rates of 23.75% for in-hospital cases, 41.55% at six months,^2^ and 8 to 48% at one year.^3^ Rapid initiation of appropriate antimicrobial therapy is therefore critical, as each hour of delay is associated with an approximately 5% increases in mortality risk.^4–6^ Current Surviving Sepsis Campaign guidelines recommend empiric broad-spectrum antibiotics administration within 1 h of diagnosis, based on the most likely pathogens, infection site(s), local pathogen spread, and risks of resistant organisms.^4^

While this empiric strategy aims to minimize the risk of undertreatment, clinical data indicate substantial overtreatment with last-resort broad-spectrum antibiotics, despite a relatively low prevalence of resistant pathogens.^7^ This therapeutic misalignment not only increases mortality risk^8^ but also accelerates the emergence of antimicrobial resistance,^9–12^ highlighting the need for rapid antimicrobial susceptibility testing (AST) protocols^13–15^ to enable timely, targeted therapy.

Standard clinical microbiological diagnostics follow a two-step process defined by Clinical and Laboratory Standards Institute (CLSI) guidelines: species identification (ID), followed by AST to assess antibiotic susceptibility. Although culture-based methods provide reliable sensitivity and specificity, they typically require 48–72 h and involve extensive workflows,^13^ as summarized in Figure 1 (lower panel). Recent advances, including the rapid AST protocol developed by the European Committee on Antimicrobial Susceptibility Testing (EUCAST)^16,17^ and molecular approaches such as polymerase chain reaction (PCR) and next-generation sequencing (NGS), have improved turnaround time.^13,15,18^ However, EUCAST rapid AST relies on preliminary species ID for breakpoint interpretation, while molecular methods detect resistance genes and may not reflect phenotypic susceptibility under clinical conditions.^19^

**Figure 1:**
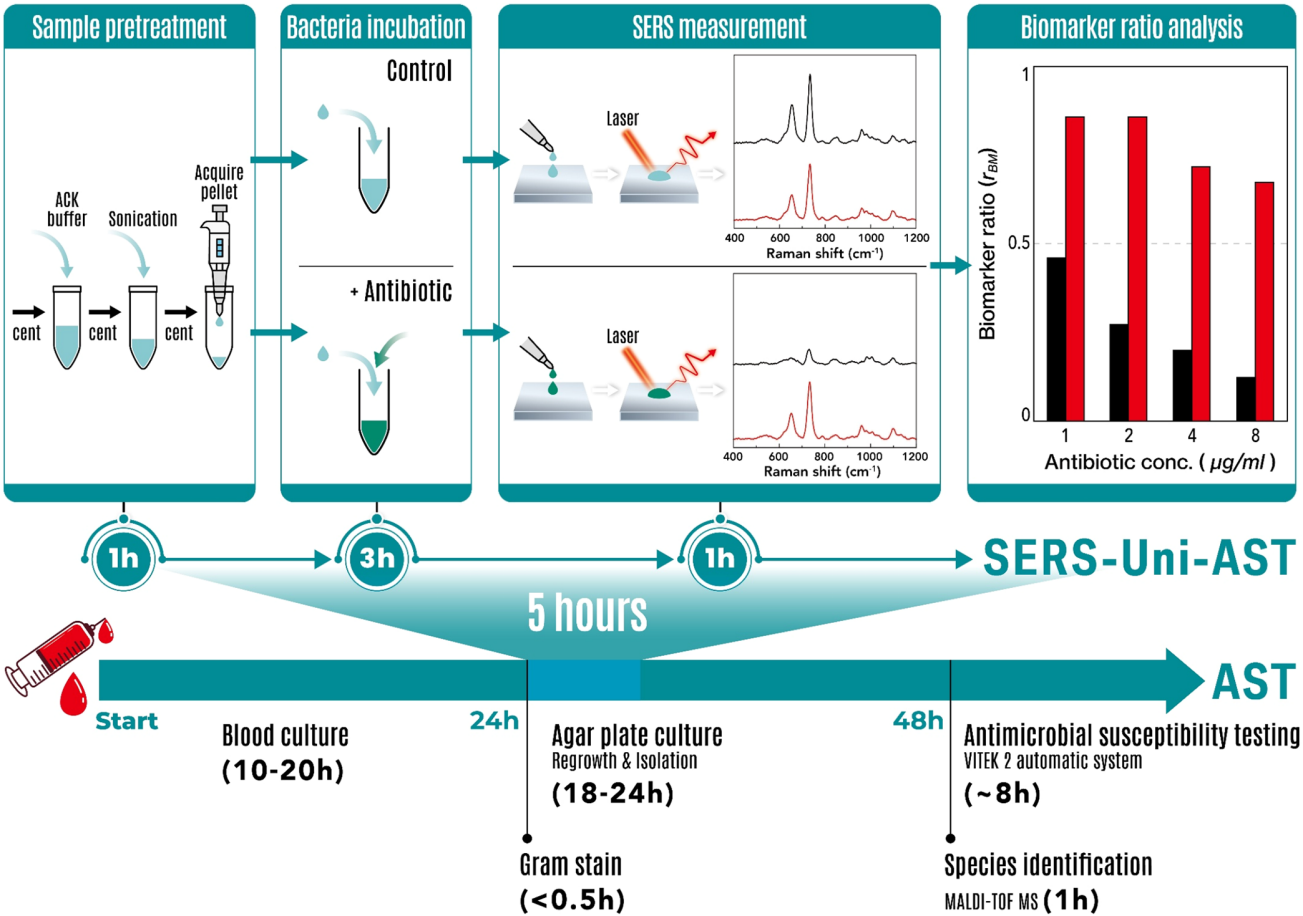
Comparative timeline of SERS-Uni-AST and conventional antimicrobial susceptibility testing (AST). **Upper panel:** The SERS-Uni-AST workflow provides results within 5 h after Gram staining, including sample pretreatment with ammonium–chloride–potassium (ACK) buffer and sonication (1 h), bacterial incubation with and without antibiotics (3 h), and SERS measurement with biomarker ratio analysis (1 h). Representative SERS spectra illustrate differences between susceptible (black) and resistant (red) isolates; the corresponding biomarker ratios (*r*_BM_), defined as the biomarker intensity in the antibiotic-treated condition normalized to the paired control, is plotted versus antibiotic concentration. **Lower panel:** The conventional AST workflow after Gram staining comprises agar plate subculture (18–24 h), species identification by matrix-assisted laser desorption/ionization time-of-flight mass spectrometry (MALDI-TOF MS, ∼1 h), and automated susceptibility testing using VITEK 2 (∼8 h).

Phenotypic AST remains the clinical standard for directly assessing bacterial growth inhibition at defined antibiotic concentrations and closely correlates with treatment outcomes. However, its reliance on species-specific minimum inhibitory concentration (MIC) breakpoints—rigorously defined in clinical guidelines based on organism-specific resistance mechanisms and epidemiological data^20–23^ —necessitates prior species identification, substantially prolonging turnaround time. Developing a phenotypic AST that bypasses species identification would be a major yet unresolved, challenge.

Additionally, early bacterial response measurements, such as inhibition zone formation in agar media or optical density changes in broth, are often unreliable during the initial incubation period,^24–26^ necessitating prolonged culture times to achieve accurate and reproducible susceptibility results.^17^ This limitation is partly attributable to antibiotic-induced morphological variations, including elongation and filamentation,^27–31^ as well as heterogeneous growth responses associated with different antibiotic mechanisms of action.^32^ Alternative approaches including microfluidic single-cell analysis and bacterial labeling methods have been developed to enhance detection sensitivity.^33–37^ However, these methods typically rely on complex instruments, sequential measurements, or species-specific labeling molecules, limiting their scalability and routine clinical adoption. Collectively, these constraints underscore the urgent need for rapid AST protocols that employ the sensitive, label-free detection of bacterial phenotypic responses.

Surface-enhanced Raman scattering (SERS) is a promising label-free analytical technique for rapid microbial diagnosis.^38–41^ By leveraging signal enhancement from localized electromagnetic fields generated at metallic nanoparticle surfaces,^42^ SERS achieves high sensitivity in capturing bacterial vibrational fingerprints. Prior mechanistic and metabolomic studies have demonstrated that the characteristic bacterial SERS features can be reproduced using cell-free supernatant and quantitatively attributed to a small set of purine derivatives, rather than macromolecular components of the bacterial cell surface.^43,44^ By amplifying Raman signals predominantly from extracellular purine metabolites released during bacterial metabolic activity, SERS enables highly sensitive detection of bacterial physiological responses. Despite its potential, SERS remains sensitive to background interference, substrate heterogeneity, and sensitivity to measurement conditions—factors that are particularly critical for quantitative applications such as AST.

Building on iterative development and validation across reference strains, clinical isolates, and freshly positive blood-culture specimens since 2011, we previously established a SERS-based AST (SERS-AST) framework capable of reporting susceptibility results.^45–47^ In these prior studies, SERS measurements were performed on a Raman-enhancing AgNP/AAO substrate (a silver nanoparticle array grown within anodic aluminum oxide nanochannels), which provides stable, spatially uniform enhancement suitable for quantitative analysis of antibiotic-induced changes in purine-associated biomarker signals.^52^ In addition, hemoglobin-associated spectral interference from blood-culture samples was minimized by a standardized pretreatment protocol (ACK-buffer lysis with sonication and washing),^48^ and susceptibility discrimination was performed using receiver operating characteristic (ROC) analysis.^46,47^ Under this integrated workflow, SERS-AST achieved categorical agreement rates of 96% for Gram-positive cocci (GPC) and 97% for Gram-negative bacilli (GNB) compared with the clinical standard VITEK 2 system across eight blood-culture bacterial species and seven antibiotics within 5 h,^47^ paving the way for subsequent advances toward species-independent AST.

In this study, we present SERS-Uni-AST (Figure 1, upper panel), an enhanced SERS-AST workflow designed to enable species-independent, rapid phenotypic susceptibility determination within hours directly from positive blood-culture isolates. We evaluate its performance across a clinically diverse validation cohort and assess whether purine-associated biomarker responses can support robust, concentration-tolerant susceptibility interpretation within a clinically actionable turnaround time.

## Experimental Section

### Study Design

SERS-Uni-AST was developed as an enhanced version of the established SERS-AST methodology to enable the species-independent analysis of blood-culture bacteria. This clinical validation study evaluated the platform’s performance on aerobic bacteria from monomicrobial bloodstream infections (BSIs). Clinical isolates were obtained by random sampling from positive blood-culture bottles at the Clinical Microbiology Laboratory, Department of Laboratory Medicine, National Taiwan University Hospital (NTUH), a tertiary medical center, ensuring the representation of clinically relevant BSI pathogens.

The evaluation was designed to test SERS-Uni-AST across clinically relevant isolates against seven commonly prescribed antibiotics, selected according to Gram classification: oxacillin (OXA), ampicillin (AMP), vancomycin (VAN), and levofloxacin (LVX) for GPC cefotaxime (CTX), ceftazidime (CAZ), imipenem (IPM), and LVX for GNB. The laboratory workflow comprised three sequential steps: (1) sample pretreatment, (2) bacterial incubation with antibiotics, and (3) SERS measurement (Figure 2) followed by biomarker ratio analysis.

**Figure 2:**
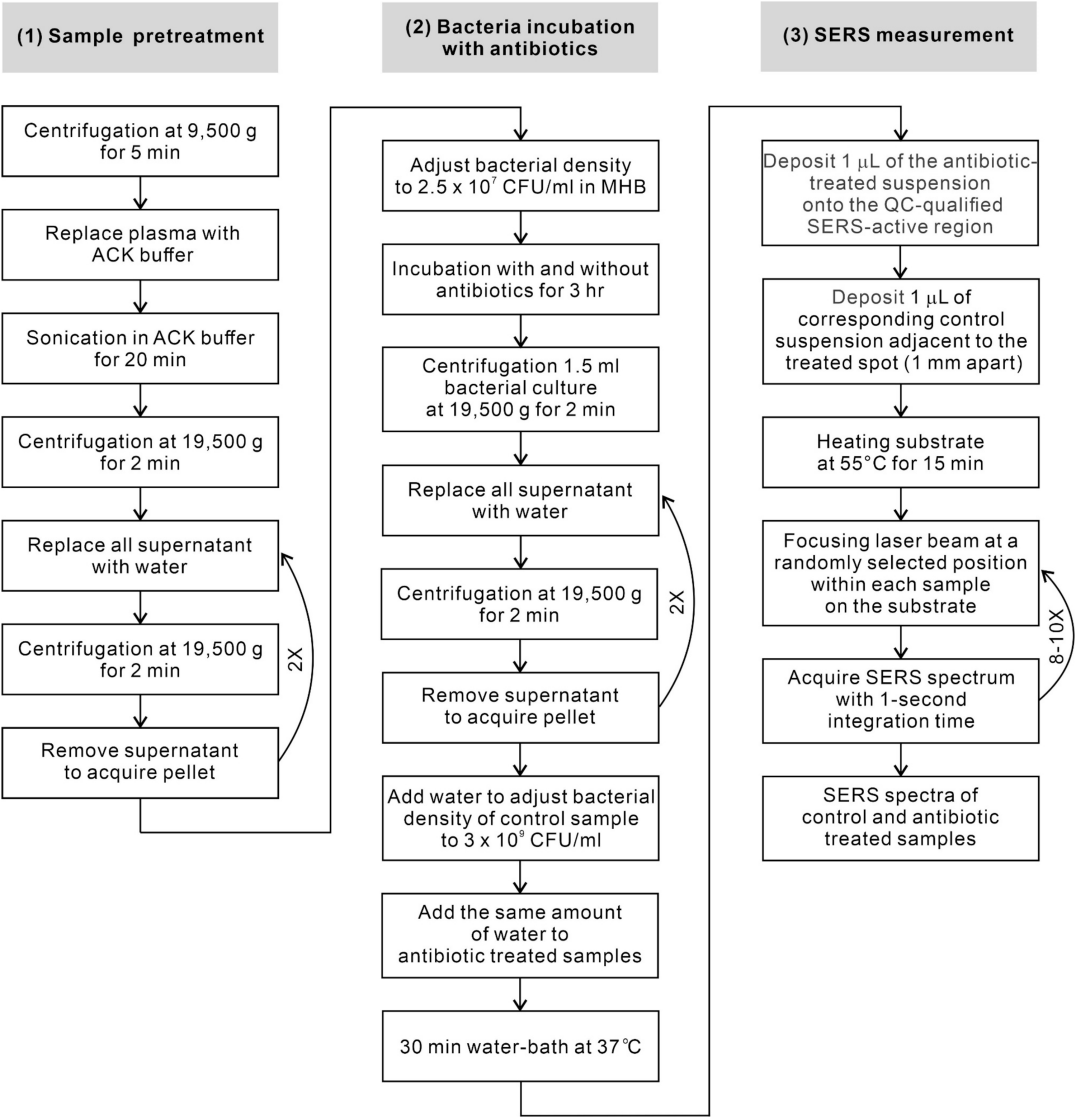
SERS-Uni-AST laboratory workflow for rapid, species-independent antibiotic susceptibility determination. The three-stage workflow processes positive blood-culture isolates through: (1) sample pretreatment to remove blood-associated interference, (2) bacterial incubation with antibiotics, and (3) SERS measurement using paired antibiotic-treated and control suspensions within the quality control (QC)-tested region of SERS substrate.

The VITEK 2 automated system (bioMérieux, Hazelwood, Missouri, USA), the clinical standard at NTUH, served as the reference method for susceptibility testing, while bacterial species ID was obtained by matrix-assisted laser desorption/ionization time-of-flight mass spectrometry (MALDI-TOF MS; Bruker Daltonics, Billerica, MA, USA) through a routine clinical workflow. In this study, species ID was not used for SERS-Uni-AST analysis and was withheld during testing; it was revealed only at the final validation stage for post hoc comparison with the corresponding VITEK 2 results.

To assess the reproducibility, the study design emphasized biological replication rather than repeated technical measurements. Each isolate obtained from an independent positive blood-culture sample was treated as an independent biological replicate, and multiple isolates were included for clinically prevalent species whenever available.

### Ethical Approval Statement

This study was conducted in accordance with the Declaration of Helsinki and approved by the Research Ethics Committee of NTUH (NTUH-REC 202111071W) with exempt status. All patient-related data were fully deidentified to ensure confidentiality throughout the study.

### Sample Pretreatment

From positive blood-culture bottles, 5 mL of broth was collected after Gram-stain classification but prior to a conventional agar subculture. Hemoglobin-associated spectral interference was removed by treating samples with ammonium–chloride–potassium (ACK) buffer for red blood cell lysis,^48^ followed by ultrasonication to induce sonoporation.^49^ After centrifugation, bacterial pellets were resuspended in Mueller–Hinton broth (MHB) and standardized to 2.5 × 10^7^ CFU/mL based on optical density measurements at 600 nm (OD_600_). The standardized bacterial suspensions were subsequently subjected to antibiotic exposure at predetermined concentrations.

### Bacterial Incubation with Antibiotics

Stock solutions (1 μg/mL) of seven lyophilized antibiotics (Merck KGaA) were prepared using manufacturer-recommended solvents. Multiple testing concentrations were selected based on CLSI guidelines^23^ and institutional antimicrobial surveillance data from NTUH to ensure coverage of concentrations spanning one dilution below and above the clinical breakpoint for commonly encountered pathogens (Table S1, Supporting Information). For GPC isolates, the tested concentrations were OXA (0.25, 0.5, 2, and 4 μg/mL), AMP (0.25, 2, 4, 8, and 16 μg/mL), VAN (2, 4, 8, 16, and 32 μg/mL), and LVX (1, 2, 4, and 8 μg/mL). For GNB isolates, the tested concentrations were CTX (1, 2, 4, 8, 16, 32, and 64 μg/mL), CAZ (4, 8, 16, and 32 μg/mL), IPM (1, 2, 4, 8, and 16 μg/mL), and LVX (1, 2, 4, and 8 μg/mL).

Each bacterial isolate was cultured in multiple tubes containing 2 mL of MHB with calculated volumes of antibiotic stock solutions to achieve the target concentrations, resulting in 18 tubes for GPC isolates and 20 tubes for GNB isolates, along with one antibiotic-free control per isolate. After incubation at 37 °C for 3 h to maximize metabolic differences between antibiotic-treated and untreated conditions,^50^ bacterial cells were pelleted and resuspended in deionized water. Control samples were adjusted to a final concentration of 3.0 × 10^9^ CFU/mL, and antibiotic-treated samples were resuspended using equivalent volumes. All suspensions then underwent an additional incubation step in a shaking water bath (37 °C, 30 min) to enhance SERS signal intensity by increasing outer membrane permeability and hence facilitating purine derivative release.^47,51^ This step, originally introduced for *Acinetobacter baumannii*,^47^ was applied uniformly to all isolates to maintain a standardized, species-blinded workflow.

### SERS Measurement

SERS substrates were fabricated as previously described.^52^ Briefly, aluminum-coated glass slides were anodized in sulfuric acid to create hexagonally packed nanochannels, followed by electrochemical plating to grow silver nanoparticles (30–50 nm in diameter with 5–10 nm spacing), yielding a 6.5 × 2.5 cm SERS-active area. Substrate performance was first qualified using a 10^–4^ M adenine test solution, and only substrates showing <15% signal variation across the active area were accepted for experiments. For each accepted substrate, a rapid mapping procedure was then performed to identify a quality control (QC)-tested region with comparable enhancement performance; all sample suspensions were deposited within this region to minimize spatial variability due to substrate heterogeneity. For each isolate–antibiotic concentration condition, the antibiotic-treated suspension was deposited onto this QC-tested SERS-active region alongside the corresponding control suspension (1 mm separation, to control local substrate variation for paired comparison) and dried at 55 °C for 15 min to form ∼1.5 mm-diameter spots.

Bacterial SERS measurements were performed using a 632.8 nm He–Ne laser system integrated with an upright microscope (BX61WI, Olympus), a Raman probe (Superhead, Horiba), a spectrometer (HE 633, Horiba), and a cooled CCD camera (Synapse, Horiba). Using a 20× objective lens, spectra were collected at 20 cm^–1^ resolution with ±3 cm^–1^ accuracy. For each dried spot, spectra were acquired at ten randomly selected, spatially distinct laser interrogation locations and averaged to represent the SERS response for the corresponding isolate condition.

Background fluorescence and cosmic ray artifacts were removed by using a nonlinear iterative peak-clipping algorithm as a standardized preprocessing step. Spectral quality control was applied by using predefined, quantifiable criteria rather than subjective visual inspection. Specifically, spectra were required to exhibit the characteristic bacterial SERS feature near 724–730 cm^–1^ and a signal-to-noise ratio (SNR) >3, where SNR was calculated as the peak intensity divided by the standard deviation (σ) of a nearby baseline window (800–900 cm^–1^). Spectra failing to meet these criteria, due to irregular peak shapes, excessive background, or insufficient SNR, were excluded from further analysis. Representative examples of accepted and excluded spectra are provided in Figure S1 (Supporting Information).

### SERS Biomarker Ratio Analysis

SERS spectral analysis focused on Raman shifts within the 400–1200 cm^–1^ region. Distinctive Raman bands were adopted as bacterial SERS biomarkers, with the 730 cm^–1^ band used for GPC and the 724 cm^–1^ band used for GNB, consistent with prior assignments.^44,53^ Antimicrobial inhibitory effects were quantified using the biomarker signal ratio *r*_BM_ (i.e., *r*_730_ for GPC and *r*_724_ for GNB), defined as the intensity ratio at the biomarker band between the antibiotic-treated sample and its paired untreated control (*r*_BM_ = *S*_ν_BM__^D^/*S*_ν_BM__^0^), where ν_BM_ denotes the biomarker band, *S*_ν_BM__^D^ is the biomarker intensity in the antibiotic-treated sample, and *S*_ν_BM__^0^ is the corresponding intensity in the paired untreated control. Antimicrobial susceptibility was subsequently determined by *r*_BM_^*^, the decision threshold applied to *r*_BM_, using either a universal decision threshold (*r*_UNI_^*^) or optimized thresholds derived from ROC analysis (*r*_OP_^*^) for each bacteria–antibiotic combination at the critical concentration.

### Receiver Operating Characteristic (ROC) Analysis

ROC analysis was used to determine the optimized decision threshold (cutoff) of biomarker signal ratios (*r*_OP_^*^) for antibiotic susceptibility assessment. Reference bacterial species ID and AST data were obtained through a routine clinical microbiology workflow using MALDI-TOF MS and VITEK 2 systems. Only SERS spectral datasets with the corresponding VITEK 2 results were included in the analysis. The ROC analysis workflow (Figure S2, Supporting Information) was adapted from our previously reported method.^47^

For each antibiotic, multiple critical concentrations (*D*_C_) were selected based on breakpoint concentrations defined in CLSI AST standards for the most commonly enrolled bacterial species (Table S1, Supporting Information). Each *D*_C_ was applied uniformly to all samples within the corresponding Gram group (GPC or GNB). Susceptibility determination followed the criterion

1 $$r_{\mathrm{BM},D_{C}},\cdot \cdot \cdot ,r_{\mathrm{BM},D_{\max}}\leq r_{\mathrm{BM}}^{*}\left\{D_{C}=D_{\min},\cdot \cdot \cdot ,D_{\max}\right\}$$

where *r*_BM,*D*_C__ denotes the biomarker signal ratio at a given critical concentration (*D*_C_), *D*_min_ and *D*_max_ represent the minimum and maximum tested concentrations, respectively, and *r*_BM_^*^ is the decision threshold, ranging from 0 to the observed maximum value. Samples satisfying eq 1 criteria were classified as susceptible; others were classified as resistant.

For each bacteria–antibiotic combination, SERS-Uni-AST results were compared with VITEK 2 results to determine true positive (TP), false positive (FP), true negative (TN), and false negative (FN) outcomes. For binary classification, intermediate results reported by VITEK 2 were treated as resistant. These values were used to calculate positive percentage agreement (PPA = *n*_TP_/(*n*_TP_ + *n*_FN_)) and negative percentage agreement (NPA = *n*_FP_/(*n*_FP_ + *n*_TN_)), where *n* denotes the sample numbers in each category. ROC curves were generated by varying *r*_BM_^*^ across its observed range for different *D*_C_ values between *D*_*min*_ and *D*_max_. For each *D*_C_, the optimized threshold value *r*_OP_^*^ was determined by maximizing Youden’s index (*J* = PPA + (NPA–1)) during resistant-sample validation (Figure S3, Supporting Information).

### Validation of SERS-Uni-AST with the Clinical Standard AST Method

Categorical susceptibility results obtained by SERS-Uni-AST were compared to those generated by the VITEK 2 system. Performance was evaluated using categorical agreement (*R*_A_), major errors (false-resistant cases, *N*_ME_), very major errors (false-susceptible cases, *N*_VME_), and a weighted average of *R*_A_—calculated by weighting individual *R*_A_ values by sample size. In addition, the area under the curve (AUC) was calculated to assess the discriminative performance.

## Results and Discussion

### Sample Inclusion Workflow and Cohort Validation

Between June 2018 and February 2023, a total of 235 clinical isolates were collected from positive aerobic blood culture bottles after Gram staining and processed for SERS-Uni-AST, comprising 102 GPC and 133 GNB.

Quality control procedures led to the exclusion of 44 isolates during the validation workflow, as summarized in Figure S4 (Supporting Information). Fifteen isolates were excluded due to technical factors, including insufficient bacterial growth (*n* = 3, including one anaerobe) and inadequate SERS signal quality (*n* = 12). An additional 29 isolates were excluded after completion of the laboratory workflow because they were identified as polymicrobial infections by MALDI-TOF MS, despite initial Gram-stain results suggesting monomicrobial cultures.

The final validation cohort comprised 191 confirmed monomicrobial isolates (86 GPC and 105 GNB), encompassing 43 bacterial species (20 GPC and 23 GNB). These isolates generated 5,800 high-quality spectral datasets across seven antibiotics, with VITEK 2 results available for all included bacterium–antibiotic combinations.

The species distribution observed in enrolled monomicrobial aerobic isolates reflected the regional epidemiology of bloodstream infection at NTUH (Table[Table tbl1]). Among GPC isolates, *Staphylococcus spp*. predominated (*n* = 51), followed by *Enterococcus spp*. (*n* = 19) and *Streptococcus spp*. (*n* = 13). Among GNB isolates, *Escherichia coli* was most prevalent (*n* = 47), followed by *Klebsiella spp*. (*n* = 23), *Acinetobacter spp*. (*n* = 12), and *Pseudomonas aeruginosa* (*n* = 8). The species distribution and the associated resistance profiles over this five-year collection period were comparable to those reported by the SENTRY Antimicrobial Surveillance Program,^54^ supporting the clinical representativeness of the study cohort.

**Table 1:** Species Distribution of 205 Aerobic Isolates from Monomicrobial Bloodstream Infections (88 Gram-Positive Cocci and 117 Gram-Negative Bacilli) Analyzed in this Study^a^

Gram-positive cocci: *N*_G_ = 88		
species name	***N***_**g**_	***N***_**s**_
*Staphylococcus* spp.	51	
*Staphylococcus aureus*		18
*Staphylococcus capitis*		13
*Staphylococcus epidermidis*		8
*Staphylococcus hemolyticus*		4
*Staphylococcus hominis*		3
*Coagulase neg. staphylococci*		2
*Staphylococcus caprae*		2
*Staphylococcus cohnii subsp. urealyticus*		1
*Enterococcus* spp.	19	
*Enterococcus faecium*		13
*Enterococcus faecalis*		5
*Enterococcus avium*		1
*Streptococcus* spp.	13	
*Streptococcus dysgalactiae*		3
*Streptococcus agalactiae*		4
*Streptococcus gallolyticus*		3
*Streptococcus alactolyticus*		1
*Streptococcus sanguinis*		1
*Streptococcus mitis*		1
*Leuconostoc lactis*		2
*Micrococcus* spp.	1	
*Kocuria* spp.	1	
*Pediococcus pentosaceus*		1

Gram-negative bacilli: *N*_G_ = 118		
species name	***N***_**g**_	***N***_**s**_
*E. coli*		47
*Klebsiella pneumoniae*		22
*Klebsiella variicola*		1
*Acinetobacter* spp.	12	
*Acinetobacter baumannii* complex		7
*Acinetobacter seifertii*		2
*Acinetobacter nosocomialis*		1
*Acinetobacter ursingii*		1
*Acinetobacter soli*		1
*P. aeruginosa*		8
*Enterobacter* spp.	8	
*Enterobacter cloacae* complex		6
*Enterobacter bugandensis*		1
*Enterobacter aerogenes*		1
*Salmonella* spp.	6	
*Aeromonas* spp.	3	
*Aeromonas hydrophila*		1
*Aeromonas caviae*		2
*Proteus mirabilis*		2
*Stenotrophomonas maltophilia*		2
*Serratia marcescens*		1
*Citrobacter koseri*		1
*Shewanella putrefaciens*		1
*Burkholderia cepacia* complex		1
*Sphingomonas pseudosanguinis*		1
*Moraxella* spp.		1
*Bordetella hinzii*		1
unknown species		1

a Species identification is summarized at the genus and species levels. *N*_G_ denotes the number of isolates within each Gram-staining group, *N*_g_ denotes the number of isolates within each genus, and *N*_s_ denotes the number of isolates for each species.

Compared to our previous SERS-AST study, which evaluated eight bacterial species against seven antibiotics,^47^ this validation represents an approximately 5-fold expansion in species richness. This substantially expanded and temporally diverse cohort, including both ESKAPE pathogens and less frequently encountered species, enhances the clinical relevance and generalizability of SERS-Uni-AST as a species-independent antimicrobial susceptibility testing platform.

### Biomarker Selection and Antibiotic-Induced Spectral Responses

Figure 3 provides an intuitive illustration of the spectral basis underlying biomarker selection in the SERS-Uni-AST workflow. In the upper panel, representative spectra from GPC and GNB show pronounced treated–control differences in the purine-associated biomarker bands at ∼730 cm^–1^ (GPC) and ∼724 cm^–1^ (GNB) for susceptible isolates, whereas resistant isolates exhibit only minimal spectral change under the same conditions. These bands have been commonly assigned to vibrational modes with dominant adenine- and hypoxanthine-related contributions in GPC and GNB, respectively, as supported by prior mechanistic and metabolomic SERS studies using bacterial supernatants.^43,44^

**Figure 3:**
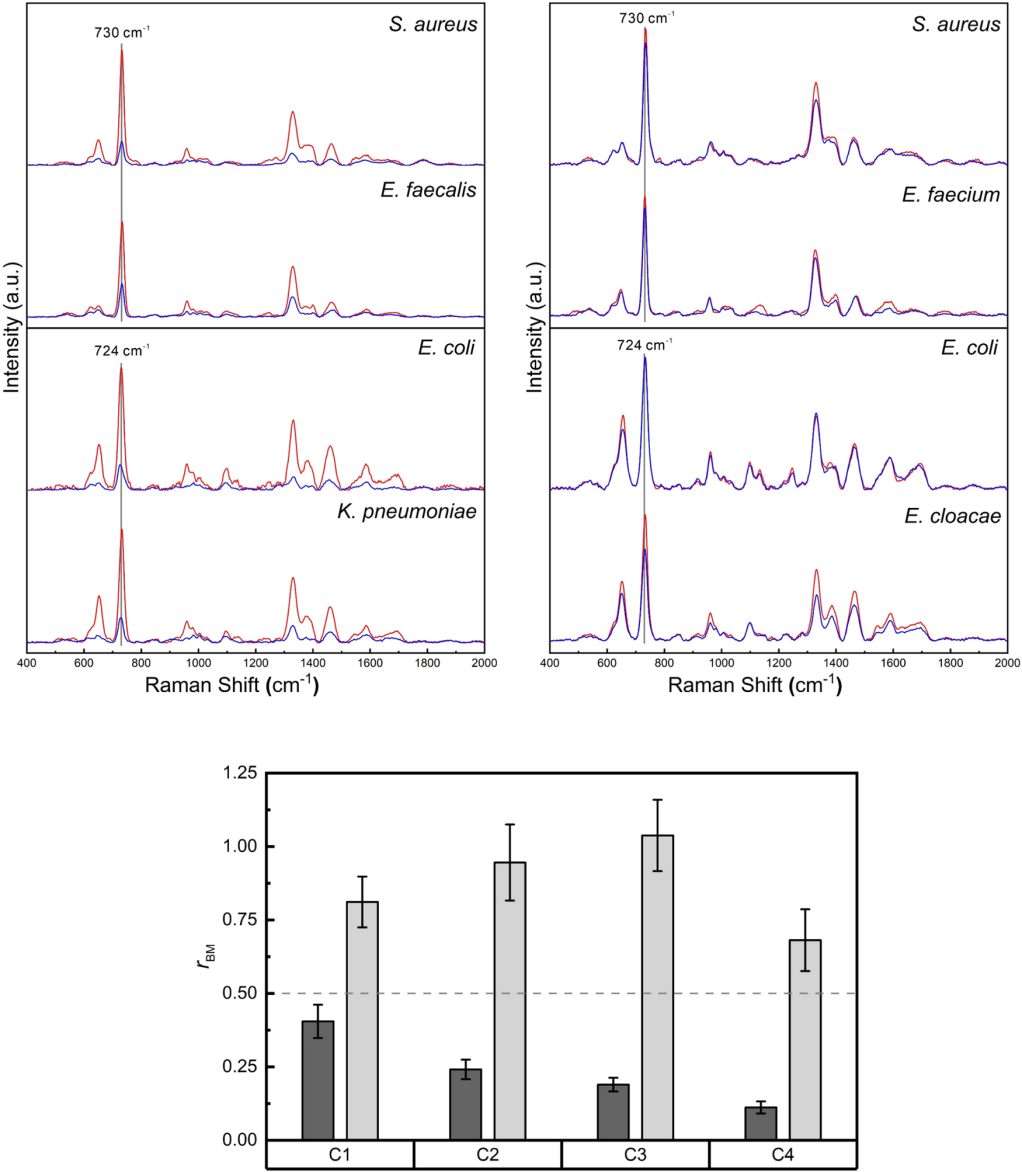
Representative SERS spectra and biomarker-based metric used for SERS-Uni-AST. Upper panel: representative overlaid SERS spectra from levofloxacin (LVX)-treated (blue) and paired control (red) conditions for a susceptible isolate (left) and a resistant isolate (right). For each susceptibility group, spectra from Gram-positive cocci (GPC; upper traces, biomarker band at 730 cm^–1^) and Gram-negative bacilli (GNB; lower traces, biomarker band at 724 cm^–1^) are shown to illustrate characteristic treated–control differences. Lower panel: example of the *E. cloacae* complex treated with LVX, showing the distribution of *r*_BM_ across concentration bins (C1–C4); C3 corresponds to the clinical breakpoint concentration. Dark gray and light gray bars denote *r*_BM_ for the susceptible and resistant isolates, respectively. The dashed line marks a reference level (*r*_BM_ = 0.5) used for comparison in subsequent analyses. *r*_BM_ is defined as the treated-to-control signal ratio at the corresponding biomarker frequency.

These qualitative spectral differences are quantitatively captured by the biomarker-based metric *r*_BM_, which summarizes the relative change in biomarker signal intensity between antibiotic-treated and paired control conditions. In the lower panel of Figure 3, the *E. cloacae* complex treated with LVX is presented as an illustrative example, showing how *r*_BM_ evolves across concentration bins (C1–C4) and how susceptible and resistant isolates follow distinct concentration-dependent trajectories, with separation becoming more apparent toward bins adjacent to the predefined critical concentration used for susceptibility classification.

To support the species-independent generalizability of these representative spectra, a cohort-level overview of bacterial SERS spectral features is provided in the Supporting Information. Representative spectra from all validated Gram-positive and Gram-negative species included in this study are shown in Figures S5 and S6. Across this taxonomically diverse set of clinical isolates, the characteristic purine-associated biomarker bands at ∼730 cm^–1^ for GPC and ∼724 cm^–1^ for GNB were consistently observed, supporting their selection as robust, species-independent SERS biomarkers for downstream susceptibility analysis.

Together, these representative spectra establish the spectral and quantitative foundation for subsequent cohort-level evaluation of antimicrobial susceptibility.

### SERS Biomarker Analysis for Antimicrobial Susceptibility Determination

To extend the representative spectral observations to the full validation cohort of 191 confirmed monomicrobial isolates, *r*_BM_ was systematically analyzed across all tested isolates, antibiotics, and concentrations. For GPC, the ratio at 730 cm^–1^ (*r*_730_) was used, whereas for GNB, the ratio at 724 cm^–1^ (*r*_724_) was applied.

Within the validation cohort, susceptible isolates consistently exhibited pronounced reductions in *r*_BM_ values upon antibiotic exposure, whereas resistant isolates maintained *r*_BM_ values near unity, reflecting preserved metabolic activity under antibiotic challenge (Figures 4 and 5). Across multiple bacterium–antibiotic combinations, the resulting *r*_BM_ distributions showed clear separation between susceptible and resistant populations over different antibiotic classes and concentration ranges, establishing a robust cohort-wide basis for categorical susceptibility interpretation.

**Figure 4:**
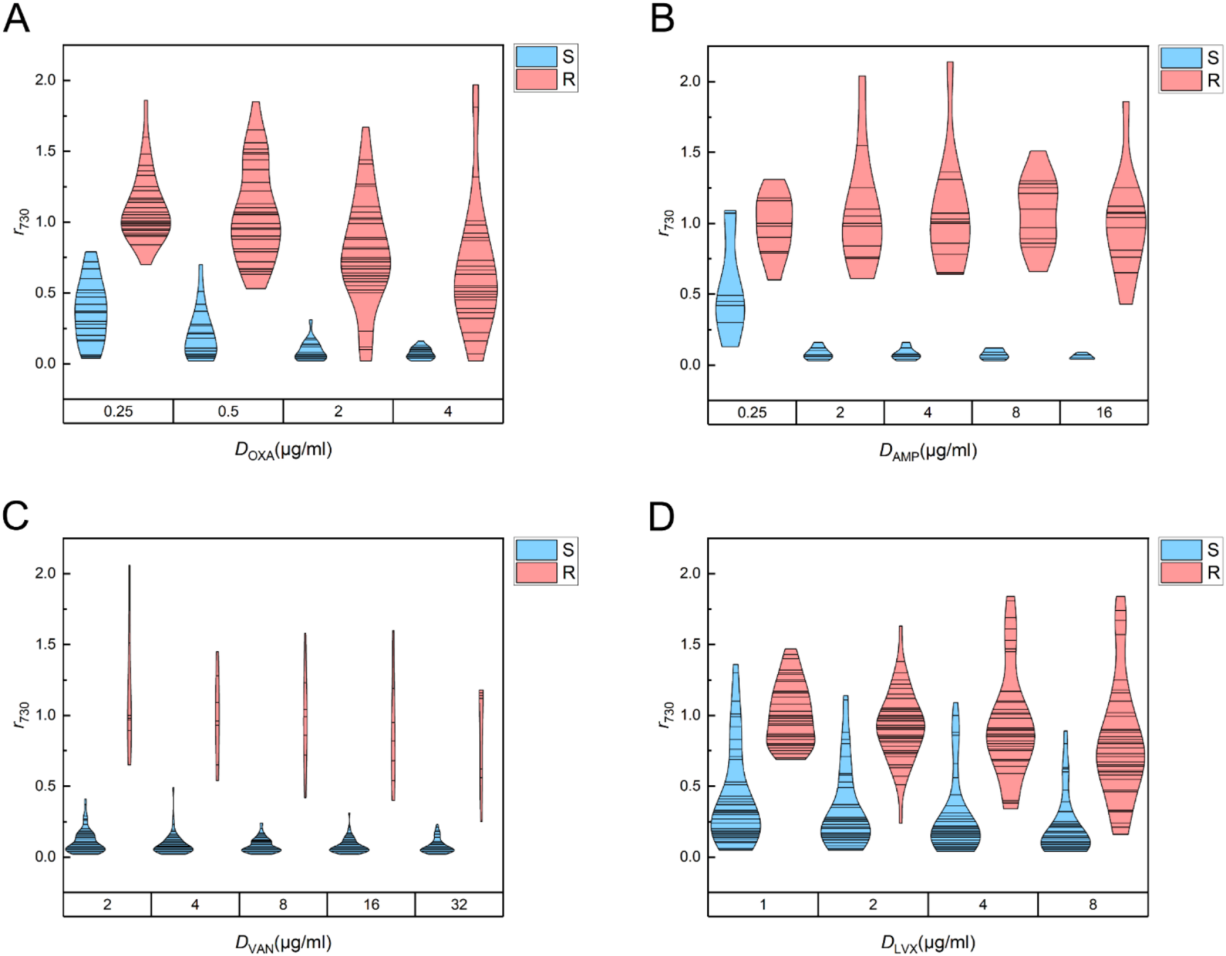
Distributions of SERS biomarker ratios (*r*_730_) for antibiotic-treated Gram-positive cocci. Violin plots show *r*_730_ values for samples treated with (A) oxacillin (OXA), (B) ampicillin (AMP), (C) vancomycin (VAN), and (D) levofloxacin (LVX) across the tested concentrations. Here, *r*_730_ is defined as the ratio of the SERS intensity at 730 cm^–1^ between the antibiotic-treated sample and its paired untreated control. Isolates are colored by VITEK 2 reference classification (S, susceptible, blue; R, resistant, red). Violin widths represent kernel density estimates (Scott bandwidth), with individual data points overlaid.

**Figure 5:**
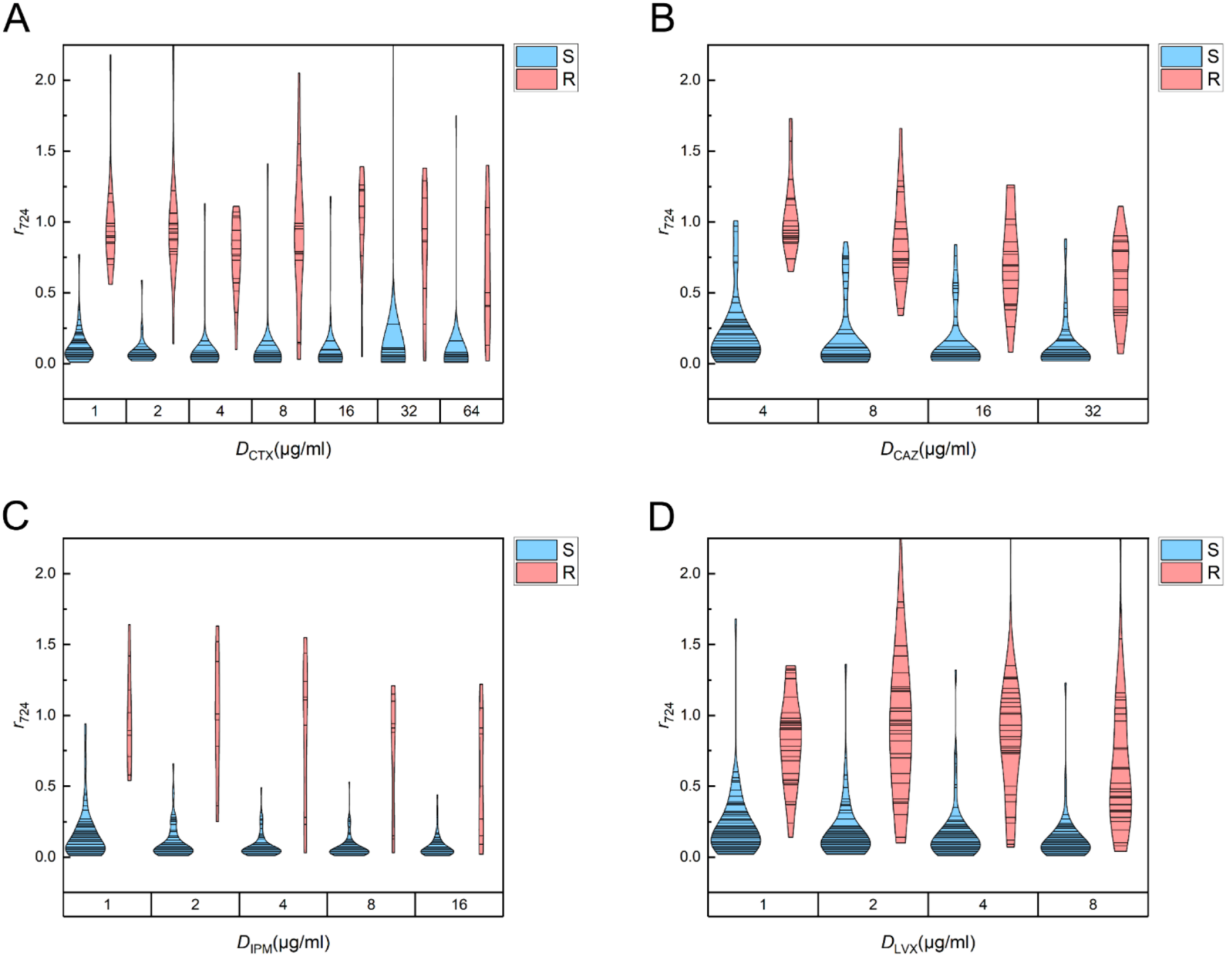
Distributions of SERS biomarker ratios (*r*_724_) for antibiotic-treated Gram-negative bacilli. Violin plots show *r*_724_ values for samples treated with (A) cefotaxime (CTX), (B) ceftazidime (CAZ), (C) imipenem (IPM), and (D) levofloxacin (LVX) across the tested concentrations. Here, *r*_724_ is defined as the ratio of the SERS intensity at 724 cm^–1^ between the antibiotic-treated sample and its paired untreated control. Isolates are colored by VITEK 2 reference classification (S, susceptible, blue; R, resistant, red). Violin widths represent kernel density estimates (Scott bandwidth), with individual data points overlaid.

Beyond this population-level separation, susceptible isolates generally exhibited a progressive decline in *r*_BM_ with an increasing antibiotic concentration, whereas resistant isolates showed only modest changes. This dose–response contrast was particularly evident for AMP, VAN, CTX, and IPM (Figures 4B,C and 5A,C), where susceptible isolates predominantly exhibited *r*_BM_ values below 0.5, while resistant isolates remained above this threshold. In addition, susceptible isolates exposed to AMP showed a marked reduction in *r*_BM_ at 2 μg/mL (Figure 4B), which closely aligns with the typical MIC range of AMP for most enrolled Gram-positive pathogens, suggesting that SERS biomarker responses may capture antibiotic-specific inhibitory effects at clinically relevant concentrations.

### Categorical Agreement with Clinical Reference AST

The central innovation of SERS-Uni-AST is a species-independent interpretation strategy that applies a single decision threshold to the biomarker ratio (*r*_BM_) across bacterial species. To evaluate the feasibility of a universal decision threshold (*r*_UNI_^*^) for antimicrobial susceptibility determination, we analyzed each Gram-type antibiotic test at its selected critical concentration and compared SERS-Uni-AST results with those from the VITEK 2 system, the clinical standard automated AST method at NTUH.

Across bacterium–antibiotic combinations, agreement rate (*R*_A_) curves versus decision threshold applied to *r*_BM_ showed consistently high performance within the range *r*_BM_^*^ = 0.4–0.6 (Figure 6), and *r*_BM_^*^ = 0.5 was identified as the optimal universal decision threshold (*r*_0.5_^*^). Using *r*_0.5_^*^, the GPC–AMP, GPC–VAN, GNB–CTX, and GNB–IPM tests achieved *R*_A_ values exceeding 95% (Figure 6B,C,E,G), whereas modestly lower *R*_A_ values were observed for GPC–OXA, GPC–LVX, GNB–CAZ, and GNB–LVX (Figure 6A,D,F,H). Overall, the universal decision threshold strategy using *r*_0.5_^*^, independent of bacterial species, achieved a weighted average *R*_A_ of 92% for both GPC and GNB (Figure 6I and Table 2).

**Figure 6:**
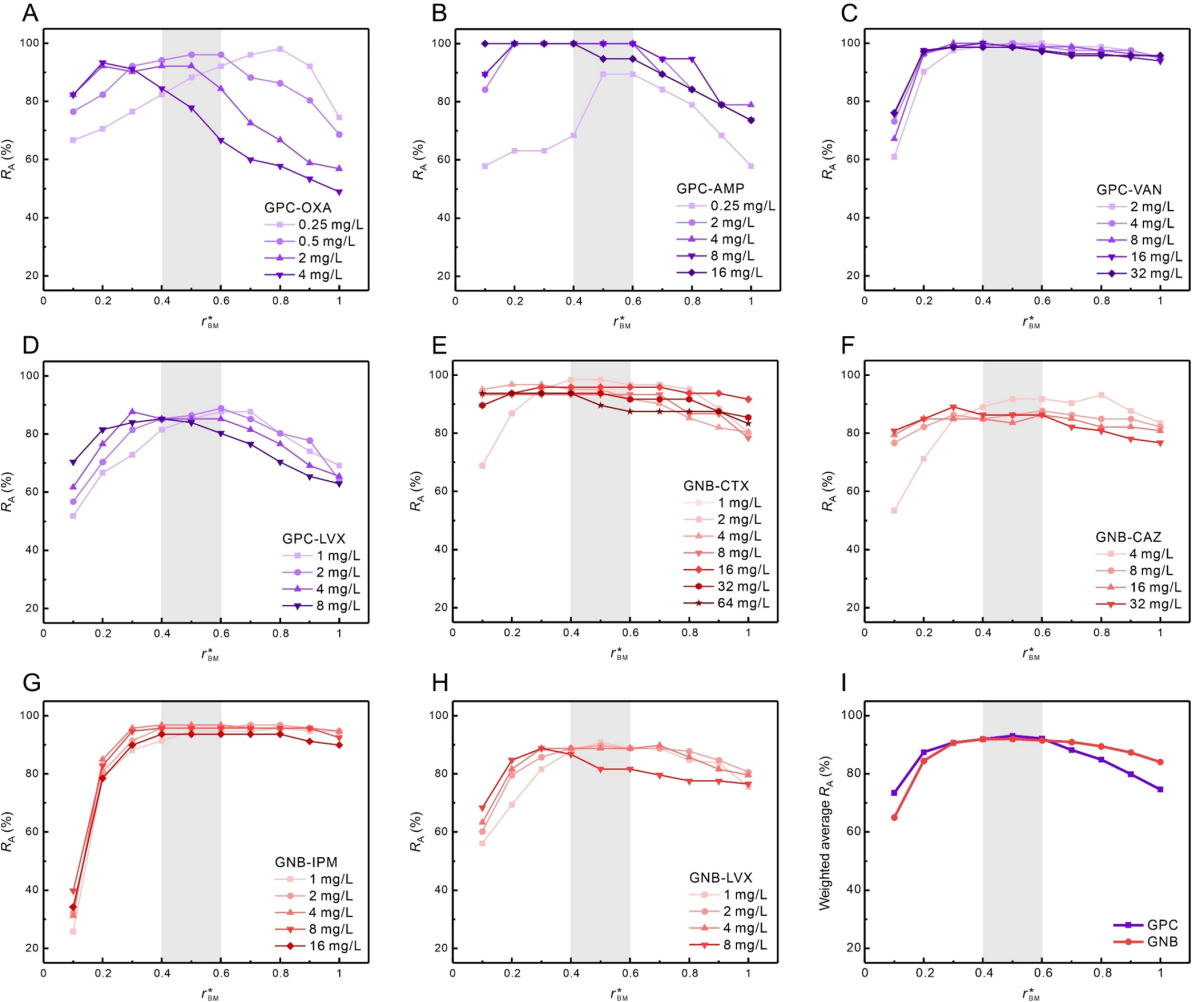
Agreement rate versus decision threshold applied to *r*_BM_ for SERS-Uni-AST. Agreement rate (*R*_A_) between SERS-Uni-AST and VITEK 2 classifications is plotted as a function of the decision threshold applied to *r*_BM_ (*r*_BM_^*^) for eight bacterium–antibiotic tests at their selected critical concentrations: (A) GPC-OXA, (B) GPC-AMP, (C) GPC-VAN, (D) GPC-LVX, (E) GNB-CTX, (F) GNB-CAZ, (G) GNB-IPM, and (H) GNB-LVX. (I) Weighted averag*e R*_A_ versus *r*_BM_^*^ for GPC and GNB groups. The gray-shaded region denotes the tolerance range (*r*_BM_^*^ = 0.4–0.6) supporting selection of a universal decision threshold. Weighted average *R*_A_ represents the overall agreement rate, calculated as the average of all *R*_A_ values weighted by the corresponding sample sizes. Abbreviations: GPC, Gram-positive cocci; GNB, Gram-negative bacilli; OXA, oxacillin; AMP, ampicillin; VAN, vancomycin; LVX, levofloxacin; CTX, cefotaxime; CAZ, ceftazidime; and IPM, imipenem.

**Table 2:**
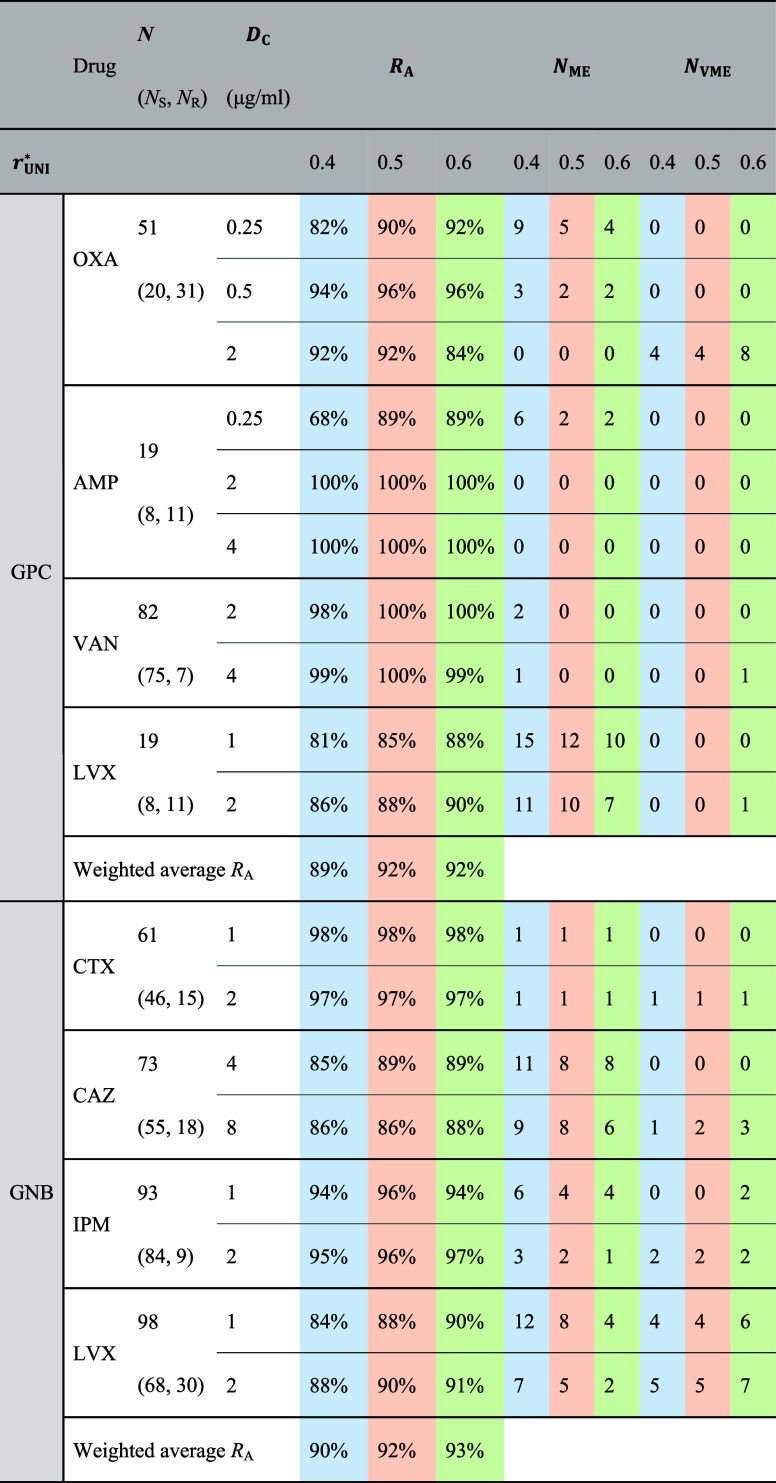
Performance of SERS-Uni-AST versus VITEK 2 Using Universal Biomarker Ratio Decision Thresholds (*r*_UNI_^*^)­^a^

a Antimicrobial susceptibility is determined by applying *r*_UNI_^*^ values of 0.4, 0.5, and 0.6 as decision thresholds to *r*_BM_ at the selected critical concentration (*D*_C_) for each bacterium–antibiotic test. Categorical agreement (*R*_A_) and error metrics are reported for each *r*_UNI_^*^. *N* denotes the total number of isolates, with *N*_S_ and *N*_R_ indicating the number of susceptible and resistant isolates as determined by VITEK 2, respectively. Major errors (*N*_ME_) correspond to false-resistant classifications, and very major errors (*N*_VME_) correspond to false-susceptible classifications. Weighted average *R*_A_ is calculated as the average of *R*_A_ values weighted by the corresponding sample sizes (*N*). Antibiotics tested: for Gram-positive cocci (GPC), oxacillin (OXA), ampicillin (AMP), vancomycin (VAN), and levofloxacin (LVX); for Gram-negative bacilli (GNB), cefotaxime (CTX), ceftazidime (CAZ), imipenem (IPM), and levofloxacin (LVX). The color shading is used exclusively to distinguish the three universal decision thresholds (*r*_UNI_^*^): 0.4 (blue), 0.5 (red), and 0.6 (green). These colors are intended for visual clarity and do not represent performance levels; all data should be interpreted based on the numerical entries.

As an alternative analysis, an optimized decision threshold derived from ROC analysis (*r*_OP_^*^) yielded weighted average *R*_A_ values of 95% for GPC and 94% for GNB, with AUC values exceeding 0.9 across all specified tests (Table S2, Supporting Information). Thus, the universal decision threshold strategy produced only a modest reduction in weighted average *R*_A_ compared with ROC-optimized cutoffs (92 vs 95% for GPC; 92 vs 94% for GNB; Tables 2 and S2), while retaining a simplified, species-independent decision rule suitable for rapid implementation.

The high-agreement performance achieved using *r*_0.5_^*^ along with its wide tolerance range is attributed to consistent bacterial biomarker detection under a standardized measurement workflow. Because SERS spectra are sensitive to substrate properties, acquisition settings, and sample handling, we controlled these factors by using standardized inoculum preparation and bacterial density, high-sensitivity, reproducible SERS substrates with QC-tested measurement regions, and elements that are critical for reliable quantitative treated versus control comparisons.

### Performance Variability and Technical Considerations

Despite these advances, ensuring consistent performance across diverse clinical species and antibiotic classes remains a significant technical challenge. While AMP, VAN, CTX, and IPM testing demonstrated excellent reliability (>95% agreement), OXA, CAZ, and LVX testing showed suboptimal but still acceptable performance (∼85–90% agreement), reflecting known biological and pharmacodynamic constraints.

Systematic evaluation revealed specific antibiotic-associated discrepancies. GPC-OXA testing exhibited critical concentration-dependent variations in categorical agreement (Figure 6A), attributable to broad species-specific breakpoint distributions (Table S1, Supporting Information). LVX testing showed reduced accuracy (Figure 6D,H) because fluoroquinolones inhibit DNA gyrase and topoisomerase IV,^55^ mechanisms that often require longer exposure times than the 3 h incubation used in this study—particularly in slow-growing organisms such as *Enterococcus* spp. and *Streptococcus* spp. Moreover, LVX’s inherently low Hill coefficient further contributes to a gradual dose–response relationship,^56–59^ limiting the reliability of early phenotypic susceptibility readout under a short incubation protocol. Similarly, CAZ testing showed reduced accuracy (Figure 6F), likely related to CTX-M β-lactamase resistance^60^ and low Hill coefficient^61^ (Table S3, Supporting Information).

Species-specific challenges were also observed. *P. aeruginosa* and *Acinetobacter* spp. (together comprising 17% of GNB isolates) exhibited distinct SERS patterns characterized by an alternative biomarker peak at 654 cm^–1^ reducing detection effectiveness when relying solely on the typical GNB 724 cm^–1^ peak band. In addition, *P. aeruginosa* showed strong fluorescent interference, while *Acinetobacter* spp. posed challenges due to slow growth and low purine secretions, further limiting the detection sensitivity.

Taken together, these antibiotic- and species-dependent variations delineate the current boundaries of the universal approach while highlighting clear directions for further protocol refinement and biomarker expansion.

### Clinical Utility and Implications

SERS-Uni-AST offers significant advantages over existing rapid AST platforms through its metabolite-based Raman spectral analysis. Unlike molecular methods, which interrogate pathogen-derived molecular targets and depend on predefined resistance markers and reference databases, this approach directly quantifies viable bacterial responses to antibiotic exposure using a universal, species-independent decision threshold.^43,45^ By comparison, although EUCAST rapid AST provides phenotypic results within 4–8 h, its accuracy is limited in early stage assessments due to antibiotic-induced bacterial morphological changes that confound colony-based measurements.^27,62^ SERS-Uni-AST overcomes this limitation by directly measuring bacterial metabolic responses, independent of morphological interference, and consistently delivers results within 5 h.

Validated across extensive and clinically representative isolate collection, SERS-Uni-AST establishes a universal, species-independent framework for antimicrobial susceptibility determination. Its robust performance across diverse bacterial species and antibiotic classes, combined with a streamlined, label-free workflow, supports potential point-of-care implementation^63^ and enables timely guidance for targeted antimicrobial therapy in the critical early phase of acute infections.

### Future Developments and Clinical Translation

Future development of SERS-Uni-AST will focus on expanding platform capabilities while addressing current technical challenges, including optimizing bacterial incubation time for antibiotics with specific mechanisms of action or low Hill coefficients (Table S3, Supporting Information).^59,61,64–67^ In parallel, broader biomarker strategies—such as leveraging full-spectrum bacterial signatures or adopting AI-assisted biomarker selection—are expected to further improve analytical accuracy and robustness.^68–70^

Beyond AST, this SERS-based platform also shows strong potential for bacterial identification. Previous studies have demonstrated successfully the identification of eight common species from clinical blood isolates when combined with deep learning methods,^69^ as well as discrimination between closely related species, such as members of the *Mycobacterium tuberculosis* complex and nontuberculous mycobacteria, using sensible functional linear discriminant analysis.^71^ The dual capability of SERS supports a unified workflow for both bacterial ID and AST, leveraging characteristic spectral patterns^69,71–73^ together with quantified metabolic responses.^45–47^

SERS-Uni-AST has the potential to substantially impact clinical practice in infection control and antimicrobial stewardship by enabling early targeted antibiotic selection. For clinicians managing life-threatening infections such as sepsis, this method provides timely susceptibility information that supports early evaluation of initial empiric antibiotic therapy and informed selection of alternative agents when needed. Such rapid turnaround is clinically critical as each hour of effective, targeted antibiotic therapy is associated with improved patient outcomes and reduces selection pressure for antimicrobial resistance. Integration with emerging technologies, such as direct bacterial capture from whole blood using synthetic peptide-coated magnetic nanoparticles,^25^ may further shorten the time from sample collection to actionable results.

## Conclusions

SERS-Uni-AST represents a significant advancement in rapid AST through a species-independent analytical framework. Using a universal decision threshold of bacterial SERS biomarker signal ratios, the method achieved a weighted average categorical agreement of 92% for both GPC and GNB, delivering results within 5 h without requiring prior species identification. Validated across 43 bacterial species and 7 antibiotic classes, this approach addresses key limitations of the existing rapid AST methods while maintaining robust diagnostic performance.

By enabling earlier susceptibility-guided antibiotic adjustment, SERS-Uni-AST directly supports sepsis management and aligns with global antimicrobial stewardship initiatives, including those advocated by the World Health Organization. This validated, evidence-based platform represents an important step toward improving clinical decision making for life-threatening bloodstream infections and advancing precision antimicrobial therapy.

## Supplementary Material



## Data Availability

The datasets generated and analyzed during this study are available from the corresponding author upon reasonable request.

## Notes

Representative accepted and excluded bacterial SERS spectra based on quantitative quality-control criteria, ROC analysis workflow, optimization of biomarker decision thresholds, sample inclusion and SERS-Uni-AST validation process, grouped SERS spectra demonstrating cross-species reproducibility for Gram-positive cocci and Gram-negative bacilli, antibiotic breakpoint concentrations used in this study, detailed performance metrics of SERS-Uni-AST compared with the VITEK 2 reference method, and Hill coefficients for evaluated antimicrobial agents (PDF)
