# Supplementary material for: SERS-Based Universal AST: Rapid Treatment Guidance for Blood-Culture Bacteria before Species Identification
Source: Anal Chem. 2026 Feb 12;98(8):5989–6002. doi: 10.1021/acs.analchem.5c04833 (PMC12961638; doi:10.1021/acs.analchem.5c04833)
Supplement: Supplementary file 1 [file ac5c04833_si_001.pdf]

Supporting Information

SERS-Based Universal AST: Rapid Treatment Guidance for Blood-Culture Bacteria Before Species Identification

Authors

Yin-Yi Han[1,2*], Yu-Tsung Huang[3], Dai-Feng Li[3], Ko-Lun Chen[4], Yi Chi[4], Hsin-Mei Tsai[4], Ho-Wen Cheng[4], Juen-Kai Wang[4,5*] & Yuh-Lin Wang[4*]

Affiliations

[1]Department of Anesthesiology, National Taiwan University Hospital, Taipei, Taiwan

[2]Department of Traumatology, National Taiwan University Hospital, Taipei, Taiwan

[3]Division of Laboratory Medicine, Department of Internal Medicine, National Taiwan University Hospital, Taipei, Taiwan

[4]Institute of Atomic and Molecular Sciences, Academia Sinica, Taipei, Taiwan

[5]Center for Condensed Matter Sciences, National Taiwan University, Taipei, Taiwan

*Corresponding author. Email: YYH, yyhan@ntuh.gov.tw; JKW, jkwang@ntu.edu.tw; YLW, ylwang@pub.iams.sinica.edu.tw

Supplementary Table and Figure

Figure S1. Representative examples of accepted and excluded bacterial SERS spectra based on quantitative quality-control (QC) criteria

Figure S2. Flow chart of receiver operating characteristic (ROC) analysis for SERS-Uni-AST.

Figure S3. Optimization of signal ratio cutoff values using ROC analysis.

Figure S4. Workflow of sample selection and SERS-Uni-AST validation.

Figure S5. Grouped SERS spectra of Gram-positive cocci (GPC) species included in the validation cohort.

Figure S6. Grouped SERS spectra of Gram-negative bacilli (GNB) species included in the validation cohort.

Table S1. Antibiotic breakpoint concentrations referenced for SERS-Uni-AST testing.

Table S2. Performance of SERS-Uni-AST versus the VITEK 2 reference method using ROC-optimized decision thresholds (r^*_{OP}).

Table S3. Hill coefficients of evaluated antimicrobial agents.

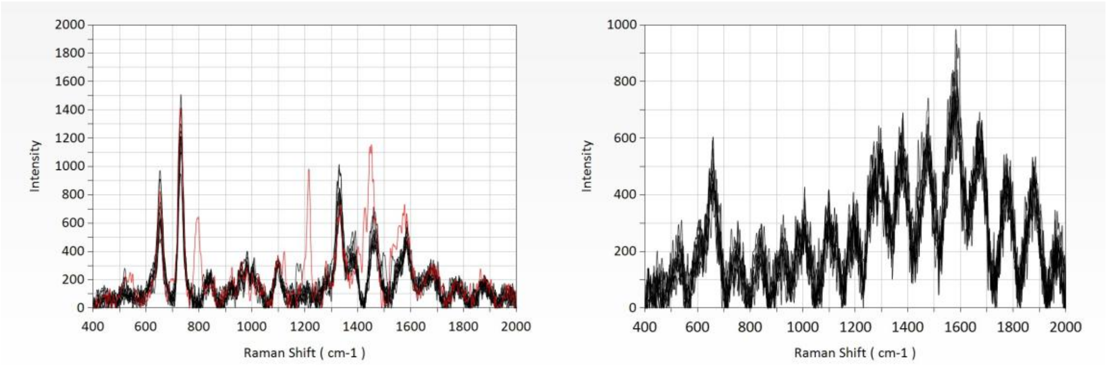

Figure S1. Figure S1. Representative examples of accepted and excluded bacterial SERS spectra based on quantitative quality-control (QC) criteria. Accepted spectra exhibit a characteristic purine-associated biomarker band near 724–730 cm^{-1} and a signal-to-noise ratio (SNR) > 3. Excluded spectra fail to meet these criteria due to insufficient SNR, excessive background, or irregular peak shapes. SNR was calculated as the peak intensity divided by the standard deviation (σ) of a nearby baseline region (800–900 cm^{-1}).

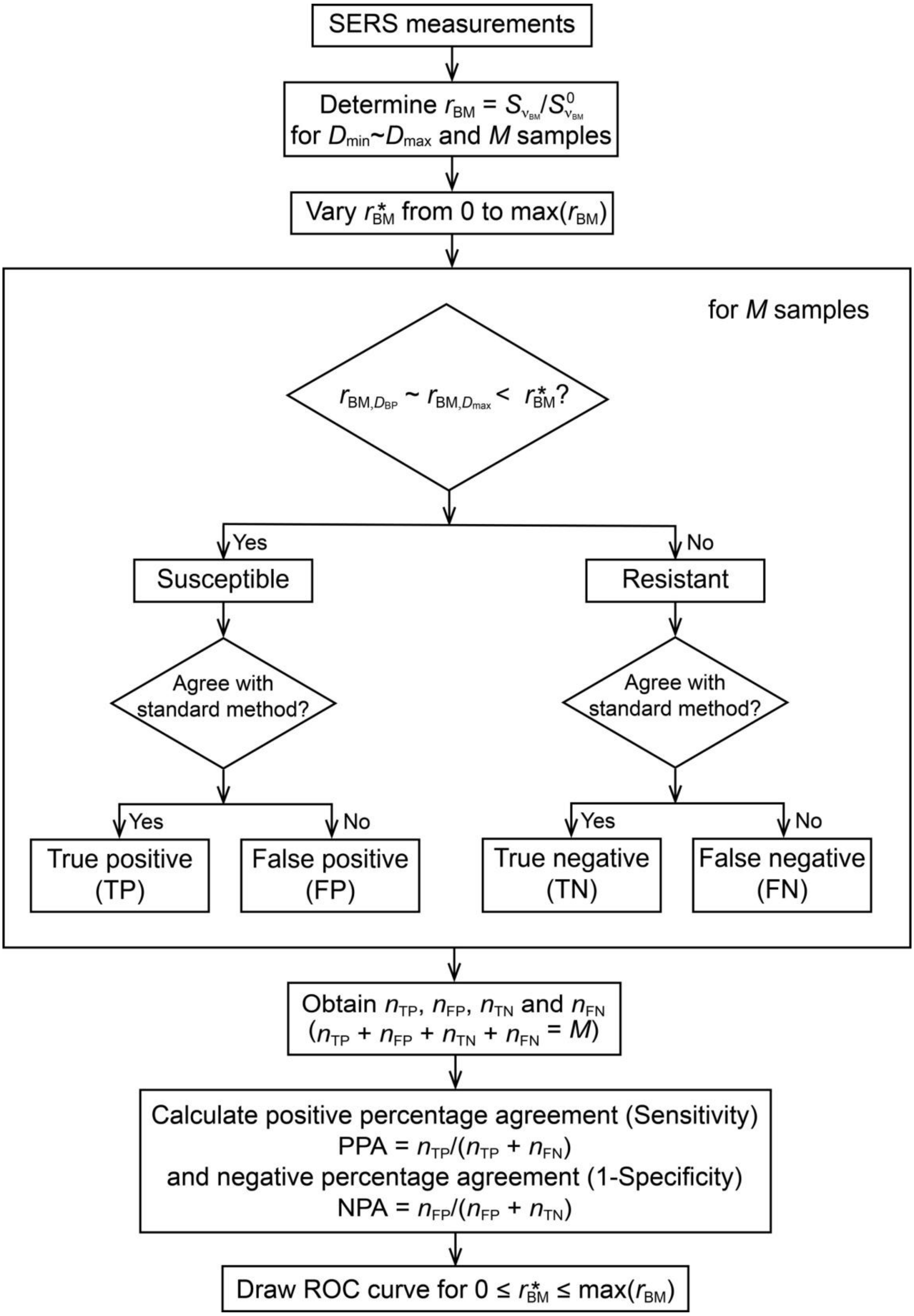

Figure S2. Flow chart of receiver operating characteristic (ROC) analysis for SERS-Uni-AST. The workflow illustrates the data analysis steps for antibiotic susceptibility determination. SERS signals at characteristic biomarker frequencies (ν_{BM}, 730 cm^{-1} for

Gram-positive bacteria and 724 cm^{-1} for Gram-negative bacteria) are measured with ($S^{\mathrm{D}}_{\nu_{\mathrm{BM}}}$) and without ($S^{0}_{\nu_{\mathrm{BM}}}$) antibiotic treatment. Signal-ratios ($r_{\mathrm{BM}} = S^{\mathrm{D}}_{\nu_{\mathrm{BM}}}/S^{0}_{\nu_{\mathrm{BM}}}$) are calculated across tested antibiotic concentrations ranging from D_{min} and D_{max}. A decision threshold (r^{*}_{BM}) is varied from 0 to maximum observed value to classify bacterial responses, which were validated against VITEK 2 reference results to determine true positive (TP), false positive (FP), true negative (TN), and false negative (FN) outcomes. The ROC curve is generated using sensitivity (positive percentage agreement (PPA $=$ $n_{\mathrm{TP}}/(n_{\mathrm{TP}} + n_{\mathrm{FN}})$) and 1-specificity (negative percentage agreement (NPA $=$ $n_{\mathrm{FP}}/(n_{\mathrm{FP}} + n_{\mathrm{TN}})$), with total sample size M = $n_{\mathrm{TP}} + n_{\mathrm{FP}} + n_{\mathrm{TN}} + n_{\mathrm{FN}}$.

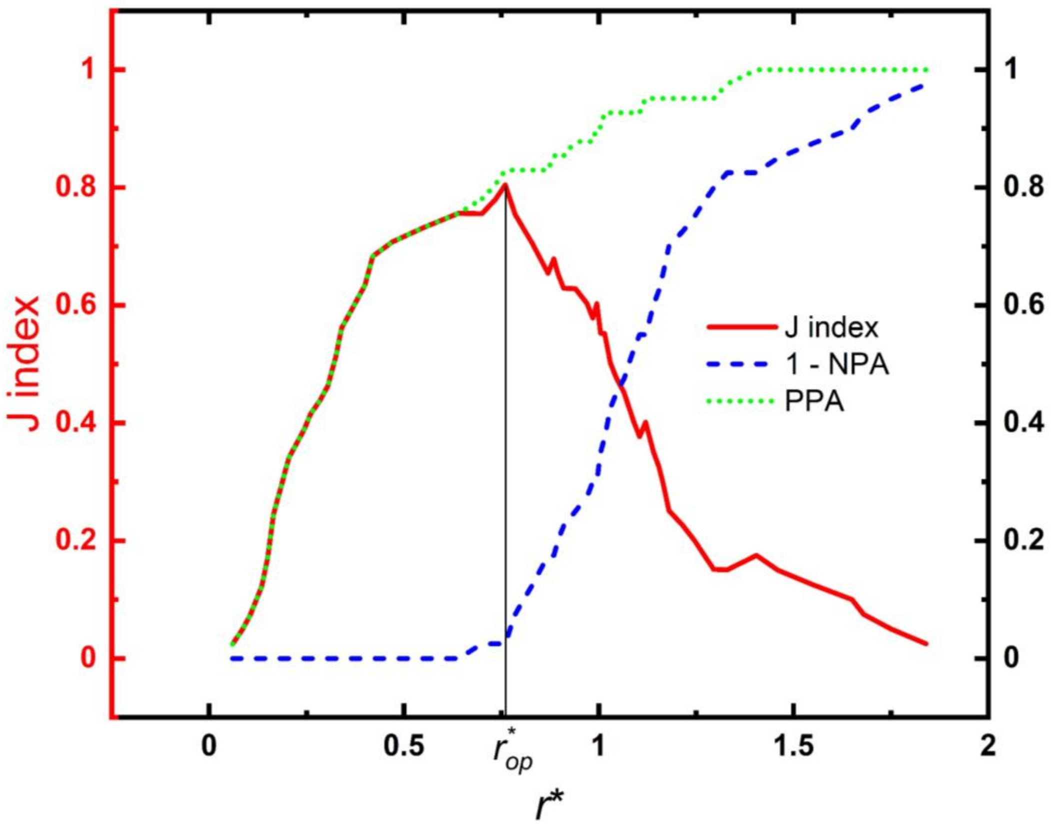

Figure S3. Optimization of biomarker signal-ratio decision thresholds **using receiver operating characteristic (ROC) analysis.** The relationships between cutoff values (r^*_{BM}) and performance metrics, including positive percentage agreement (PPA, green), negative percentage agreement (1-NPA, blue), and Youden's J index (red), are shown. The optimal cutoff (r^*_{OP}) is identified at the maximum J index value, where $J = \mathrm{PPA} + (\mathrm{NPA} - 1)$.

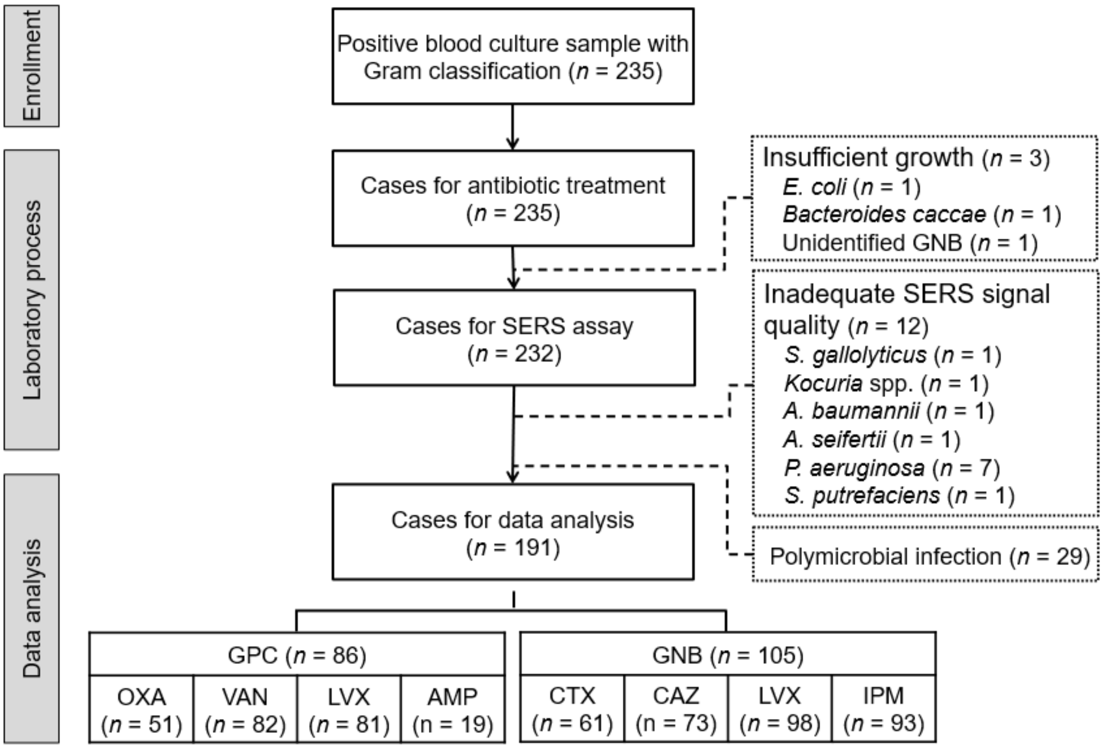

Figure S4. Workflow of sample selection and SERS-Uni-AST validation. The diagram summarizes the clinical validation process and sample exclusion criteria, indicating the number of isolates excluded at each step and the corresponding reasons.

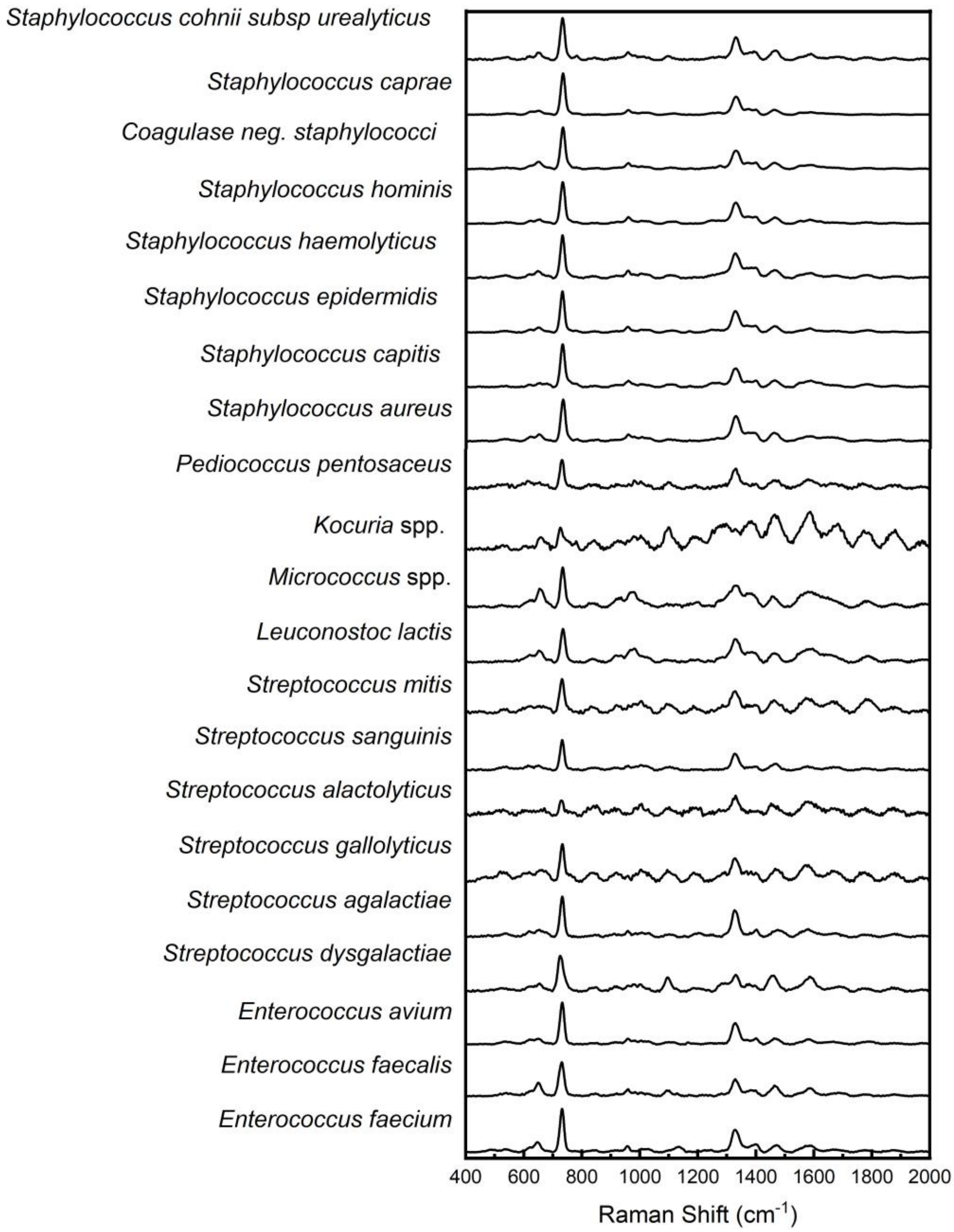

Figure S5. Grouped SERS spectra of Gram-positive cocci (GPC) clinical isolates. Representative spectra from validated GPC isolates are shown to summarize cross-species spectral features. The purine-associated biomarker band near ~730 cm^{-1} is consistently observed across isolates, supporting its selection as a robust biomarker for species-independent SERS-based AST.

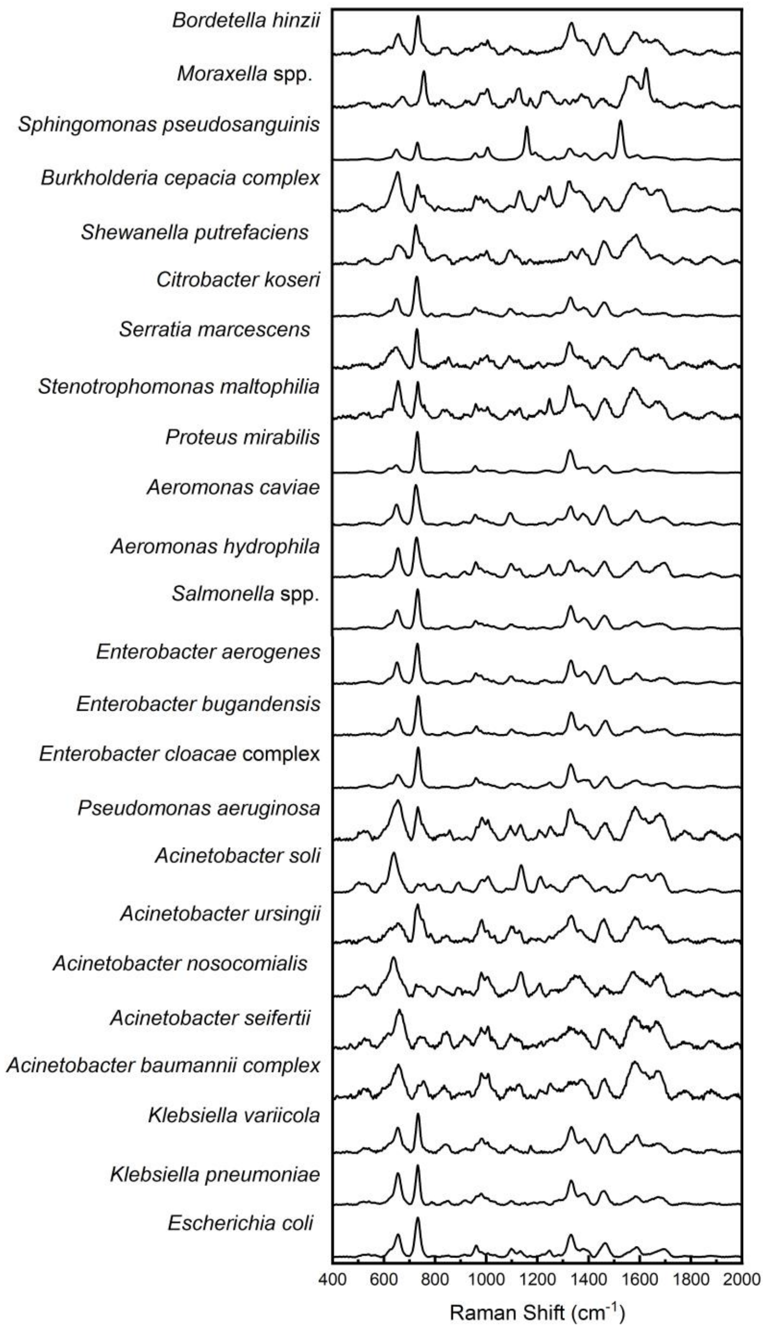

Figure S6. Grouped SERS spectra of Gram-positive cocci (GPC) clinical isolates. Representative spectra from validated GNB isolates are shown to summarize cross-species spectral features. The purine-associated biomarker band near ~724 cm^{-1} is consistently observed across isolates, supporting its selection as a robust biomarker for species-independent SERS-based AST.

Table S1. Antibiotic breakpoint concentrations referenced for SERS-Uni-AST testing. Breakpoint concentrations (μg/mL) were selected based on CLSI guidelines for Gram-positive cocci and Gram-negative bacilli and were used to define the critical concentrations and testing ranges applied in this study. Abbreviations: VAN, vancomycin; OXA, oxacillin; AMP, ampicillin; LVX, levofloxacin; CTX, cefotaxime; CAZ, ceftazidime; IPM, imipenem. S, susceptible (green); I, intermediate (yellow); R, resistant (red).

**Gram-positive cocci**	**OXA**						**AMP**							**LVX**				**VAN**				
	**0.25**	**0.5**	**1**	**2**	**4**	**8**	**0.25**	**0.5**	**1**	**2**	**4**	**8**	**16**	**1**	**2**	**4**	**8**	**1**	**2**	**4**	**8**	**16**
Staphylococcus spp.	S	R												S	I	R			S	I	R	
Coagulase negative *Staphylococcus*					S	R								S	I	R				S	I	R
Enterococcus spp.												S	R		S	I	R			S	I	R
Streptococcus pneumoniae										S	I	R			S	I	R	S (≤ 1)				
Streptococcus spp. *b*-hemolytic group							S (≤ 0.25)								S	I	R	S (≤ 1)				
Streptococcus spp. viridans group							S	I					R		S	I	R	S (≤ 1)				

**Gram-negative bacilli**	**CAZ**				**CTX**							**LVX**				**IPM**						
	**4**	**8**	**16**	**32**	**1**	**2**	**4**	**8**	**16**	**32**	**64**	**2**	**4**	**8**	**16**	**1**	**2**	**4**	**8**	**16**	**32**	**64**
Enterobacteriaceae	S	I	R		S	I	R					S	I	R		S	I	R				

Pseudomonas aeruginosa		S	I	R								S	I	R			S	I	R			
Acinetobacter spp.		S	I	R								S	I	R			S	I	R			
Stenotrophomonas maltophilia		S	I	R								S	I	R								
Burkholderia cepacian complex		S	I	R								S	I	R								
Other non-*Enterobacteriaceae*		S	I	R				S	I		R		S	I	R					S	I	R

Table S2. Performance of SERS-Uni-AST versus the VITEK 2 reference method using decision thresholds optimized by receiver operating characteristic analysis (ROC) (r^*_{OP}). For each bacterium–antibiotic test at the selected critical antibiotic concentrations (D_{C}), susceptibility was classified by comparing the biomarker signal ratio (r_{BM}) with the ROC-optimized decision threshold (r^*_{OP}). Categorical agreement rates (R_{A}), error metrics, and area under the ROC curve (AUC) are reported for each specified condition. *N* denotes the total number of isolates; N_S and N_R denote the numbers of susceptible and resistant isolates by VITEK 2. respectively. Major errors (N_{ME}) correspond to false-resistant classifications (susceptible misclassified as resistant), and very major errors (N_{VME}) correspond to false-susceptible classifications (resistant misclassified as susceptible). Weighted average R_{A} is calculated as the average of R_{A} values weighted by the corresponding sample sizes (*N*). Antibiotics tested: For Gram-positive cocci (GPC), testing included oxacillin (OXA), ampicillin (AMP), vancomycin (VAN), and levofloxacin (LVX). For Gram-negative bacilli (GNB), testing included cefotaxime (CTX), ceftazidime (CAZ), imipenem (IPM), and levofloxacin (LVX).

	**Drug**	***N*** **(*N*S, *N*R)**	**D_{C} (µg/ml)**	r^*_{OP}	R_{A}	**AUC**	N_{ME}	N_{VME}
GPC	OXA	51 (20, 31)	0.25	0.79	98%	0.99	0	1
			0.5	0.51	98%	0.99	1	0
			2	0.31	95%	0.95	0	4
	AMP	19 (8, 11)	0.25	0.49	89%	0.91	2	0
			2	0.16	100%	1	0	0
			4	0.16	100%	1	0	0
	VAN	82	2	0.49	100%	1	0	0

		(75, 7)	4	0.49	100%	1	0	0
	LVX	81	1	0.76	90%	0.92	7	1
		(41, 40)	2	0.59	90%	0.93	7	1
	Weighted average R_A: 95%							
GNB	CTX	61	1	0.38	98%	0.98	1	0
		(46, 15)	2	0.28	97%	0.97	1	1
	CAZ	73	4	0.64	90%	0.97	7	0
		(55, 18)	8	0.33	88%	0.91	9	0
	IPM	93	1	0.45	96%	0.99	4	0
		(84, 9)	2	0.34	86%	0.98	12	0
	LVX	98	1	0.56	92%	0.94	4	4
		(68, 30)	1	0.56	92%	0.94	4	4
	Weighted average R_A: 94%							

Table S3. Hill coefficients of evaluated antimicrobial agents. Hill coefficients were obtained from published pharmacodynamic studies (references in parentheses) and are provided to contextualize antibiotic-specific dose–response characteristics relevant to early susceptibility assessment in SERS-Uni-AST.

	Hill coefficient (κ)
Oxacillin	2.6 [1]
Vancomycin	3.68 [2]
Ampicillin	2.2 [1]
Levofloxacin	1.09 [3]
Cefotaxime	0.18 [4]
Ceftazidime	0.64 [5]
Imipenem	0.83 [6]

Reference

1 Lim, S. P. & Nikaido, H. Kinetic Parameters of Efflux of Penicillins by the Multidrug Efflux Transporter AcrAB-TolC of. *Antimicrob Agents Ch* **2010**, 54, 1800-1806 https://doi.org/10.1128/Aac.01714-09

2 Anderson, B. J., Allegaert, K., Van den Anker, J. N., Cossey, V. & Holford, N. H. Vancomycin pharmacokinetics in preterm neonates and the prediction of adult clearance. *Br J Clin Pharmacol* **2007**, 63, 75-84 https://doi.org/10.1111/j.1365-2125.2006.02725.x

3 Hirano, T., Yasuda, S., Osaka, Y. *et al.* Mechanism of the inhibitory effect of zwitterionic drugs (levofloxacin and grepafloxacin) on carnitine transporter (OCTN2) in Caco-2 cells. *Biochim Biophys Acta* **2006**, 1758, 1743-1750

https://doi.org/10.1016/j.bbamem.2006.07.002

4 Kjeldsen, T. S., Overgaard, M., Nielsen, S. S. *et al.* CTX-M-1 beta-lactamase expression in Escherichia coli is dependent on cefotaxime concentration, growth phase and gene location. *J Antimicrob Chemother* **2015**, 70, 62-70 https://doi.org/10.1093/jac/dku332

5 Kristoffersson, A. N., Bissantz, C., Okujava, R. *et al.* A novel mechanism-based pharmacokinetic-pharmacodynamic (PKPD) model describing ceftazidime/avibactam efficacy against beta-lactamase-producing Gram-negative bacteria. *J Antimicrob Chemother* **2020**, 75, 400-408 https://doi.org/10.1093/jac/dkz440

6 Fujimoto, M., Munakata, M. & Akaike, N. Dual mechanisms of GABAA response inhibition by beta-lactam antibiotics in the pyramidal neurones of the rat cerebral cortex. *Br J Pharmacol* **1995**, 116, 3014-3020 https://doi.org/10.1111/j.1476-5381.1995.tb15957.x
